# Retrospective rationalization of disparities between the concentration dependence of diffusion coefficients obtained by boundary spreading and dynamic light scattering

**DOI:** 10.1007/s00249-023-01664-x

**Published:** 2023-07-07

**Authors:** Donald J. Winzor, Vlad Dinu, David J. Scott, Stephen E. Harding

**Affiliations:** 1 School of Chemistry and Molecular Biosciences, University of Queensland, Brisbane, QLD 4072 Australia; 2 National Centre for Macromolecular Hydrodynamics, School of Biosciences, University of Nottingham, College Road, Sutton Bonington, LE12 5RD UK; 3 Research Complex at Harwell, OX11 OFA Rutherford Appleton Laboratory, UK

**Keywords:** Concentration dependence, Diffusion coefficient, Dynamic light scattering

## Abstract

This study establishes the existence of substantial agreement between published results from traditional boundary spreading measurements (including synthetic boundary measurements in the analytical ultracenrifuge) on two globular proteins (bovine serum albumin, ovalbumin) and the concentration dependence of diffusion coefficient predicted for experiments conducted under the operative thermodynamic constraints of constant temperature and solvent chemical potential. Although slight negative concentration dependence of the translational diffusion coefficient is the experimentally observed as well as theoretically predicted, the extent of the concentration dependence is within the limits of experimental uncertainty inherent in diffusion coefficient measurement. Attention is then directed toward the ionic strength dependence of the concentration dependence coefficient (${k}_{D}$) describing diffusion coefficients obtained by dynamic light scattering, where, in principle, the operative thermodynamic constraints of constant temperature and pressure preclude consideration of results in terms of single-solute theory. Nevertheless, good agreement between predicted and published experimental ionic strength dependencies of ${k}_{D}$ for lysozyme and an immunoglobulin is observed by a minor adaptation of the theoretical treatment to accommodate the fact that thermodynamic activity is monitored on the molal concentration scale because of the constraint of constant pressure that pertains in dynamic light scattering experiments.

## Introduction

The use of dynamic light scattering to determine the concentration dependence of the translational diffusion coefficient (*D*) for globular proteins and macromolecular assemblies (Harding and Johnson 1985a,b; Petsev and Denkov 1992; Eberstein et al. 1994; Arzenšek et al. 2012) has become standard practice because of its relative ease of use compared with the traditional boundary spreading procedures for the measurement of *D* (Cecil and Ogston 1948; Longsworth 1954; Baldwin et al. 1955; Gosting 1956; Creeth 1958). An outcome of this switch in methodology has been a changed perception of the extent of that concentration dependence (see, e.g., Harding and Johnson 1985a,b). Only slight negative concentration dependence of diffusion coefficients had been observed for globular proteins by the traditional procedure of monitoring the time-dependent spreading of an initially sharp boundary between solution and diffusate with which it is in dialysis equilibrium (Baldwin et al. 1955; Gosting 1956; Creeth 1952, 1958; Creeth et al. 1958). Indeed, the statistical uncertainty in the estimate of that concentration dependence coefficient exceeded its magnitude. On the other hand, unequivocal evidence for positive as well as negative concentration dependence of the translational diffusion coefficient has emanated from some dynamic light scattering measurements on globular protein solutions and globular macromolecular assemblies such as plant viruses (Harding and Johnson 1985a,b; Petsev and Denkov 1992; Eberstein et al. 1994; Muschol and Rosenberger 1995; Arzenšek et al. 2012; Roberts et al. 2014). This apparent difference in the concentration dependence behavior of *D* between the two approaches has gained increasing importance with the growing interest of synthetic boundary methods in sedimentation velocity analytical ultracentrifugation [see, for example, Wright et al. (2018) Schneider and Cölfen (2018), Perevyazko et al. 2021)].

This investigation extends a previous report (Scott et al. 2014) by incorporating the effects of protein net charge to improve the theoretical expression for *D*–*c* dependence in traditional diffusion experiments and thereby to justify further the earlier conclusion that the predicted slightly negative concentration dependence of *D* observed therein is encompassed by the extent of experimental scatter in diffusion coefficients obtained under the thermodynamic constraints of constant temperature and solvent chemical potential. We also establish that a slight modification of the quantitative expression for the concentration dependence of *D* from traditional diffusion coefficient measurements describes adequately the reported ionic strength dependence of ${k}_{D}\text{ obtained by dynamic light scattering}$ for lysozyme (Eberstein et al. 1994) and a monoclonal antibody (Arzenšek et al. 2012).

### Theoretical considerations

In techniques such as osmometry, sedimentation equilibrium and size exclusion chromatography, the chemical potential of the protein (species 2) is monitored under the thermodynamic constraints of constant temperature and constant solvent chemical potential (Hill 1959; Wills et al. 1993, 2015). On the grounds that small partitioning species such as buffer components and supporting electrolytes can be regarded as part of the solvent (species 1), a buffered protein solution essentially becomes a single-solute system for which the solute chemical potential, $(\mu_{2} )_{{{T,\mu_{1}} }}$, is described in terms of its standard state chemical potential, $(\mu_{2}^{0} )_{{{T,\mu_{1}} }} ,$ by the relationship

1 $$\frac{{(\mu_{2} )_{T,\mu_{1}} - (\mu_{2}^{0} )_{T,\mu_{1}} }}{RT} = \ln z_{2} = {\text{ln}} \;\gamma_{2} c_{2} /M_{2}$$

where $z_{2}$, the molar thermodynamic activity of solute, is logically written as the product of its weight concentration $c_{2}$ (g/mL) divided by molar mass $M_{2}$, and a dimensionless activity coefficient $\gamma_{2}$ to account for thermodynamic nonideality.

Because the left-hand side of Eq. (1) is also Π/(*RT*), the ratio of osmotic pressure Π to the product of the universal gas constant and absolute temperature, Eq. (1) is frequently written as the following virial expansion in ${c}_{2}$

2 $$\frac{\Pi }{RT} = \frac{{c_{2} }}{{M_{2} }} + B_{22} \left( {\frac{{c_{2} }}{{M_{2} }}} \right)^{2} + \cdots = \frac{1}{{M_{2} }}\left( {c_{2} + B_{2} M_{2} c_{2}^{2} + \cdots } \right),$$

where the second form of the expression accommodates the fact that the osmotic second virial coefficient $B_{22}$, which has the dimensions of an excluded volume (mL mol^−1^), is defined experimentally as $B_{2} = B_{22} /M_{2}^{2}$ with dimensions mL mol g.^−2^. By differentiating Eq. (2) with respect to $c_{2}$, it follows that (correct to first order in concentration)

3 $$\frac{{d[\Pi/\left( {RT} \right)]}}{{dc_{2} }} = \frac{{1 + 2(B_{22} /M_{2} )c_{2} }}{{M_{2} }} = \frac{{1 + 2B_{2} M_{2} c_{2} }}{{M_{2} }}$$

where the osmotic second virial coefficient is related to thermodynamic activity coefficient [Eq. (1)] by the relationship (Hill. 1959; Wills et al. 1993)

4 $${\text{ln}} \gamma_{2} = 2\left( {B_{22} /M_{2} } \right)c_{2} = 2B_{2} M_{2} c_{2}$$

Inasmuch as a negative gradient in solute chemical potential, $- (\partial\mu_{2} /\partial c_{2} )_{T,\mu_{1}}$, is the driving force of diffusion in traditional experiments involving a *U*-tube assembly to monitor the spreading of an initially sharp boundary between dialyzed protein solution and diffusate; the above single-solute expressions also describe the consequences of thermodynamic nonideality.

As noted by McMillan and Mayer (1945), the osmotic second virial coefficient can be related to molecular properties of the solute by the interpretation of thermodynamic nonideality on the statistical–mechanical basis of excluded volume. For example, the thermodynamic nonideality generated by a rigid uncharged sphere with radius $a_{2}$ is described by the relationship

5 $$\ln\gamma_{2} = 2B_{2} M_{2} c_{2} = \frac{{32\pi{{\text N}}_{{\text{A}}} a_{2}^{3} }}{{3M_{2} }}c_{2} + \cdots$$

where Avogadro’s number ($\text N_{\text A}$) is included to convert the excluded volume from a molecular to a molar basis (see p2435 of Harding et al 1999). In other words, the nonideality takes into account the volume from which the centers of two solute molecules are mutually excluded.

### Concentration dependence of diffusion for hard uncharged spheres

The first detailed consideration of the effect of hydrodynamic interactions on Brownian diffusion involving the net flux of solute was provided by Batchelor (1976), who developed an alternative derivation of the Einstein expression for the concentration-independent translational diffusion of molecules,

6 $$D = \frac{{{\text{k}}_{{\text{B}}} T}}{{6\pi \eta a_{2} }}$$

where the diffusion coefficient is described in terms of the Boltzman constant ${\text{k}}_{{\text{B}}} ,$ the hydrodynamic (Stokes) radius $a_{2}$ and the dynamic solvent viscosity *η*, whereas this relationship for ideal diffusion had been derived initially by Einstein on the purely thermodynamic basis of osmotic pressure, Batchelor employed a mixture of statistical mechanical and hydrodynamic considerations to achieve the same result. Adoption of that approach led to description of the concentration dependence of *D* for a rigid, uncharged spherical particles (hard sphere, HS) relative to a solvent frame of reference as

7 $$D = \frac{{\left( {1 + (8\phi - 6.55\phi} \right))k_{B} T}}{{6\pi \eta a_{2} }} = D_{o} \left[ {1 + \left(\lambda {_{T}^{HS} -\lambda _{H}^{HS} } \right)\phi } \right]$$

in which *ϕ*, the volume fraction occupied by the diffusing particles, is the product of concentration $c_{2}$ of solute and its solvated specific volume, $v_{s} = 4\pi \text N_{\text A} a_{2}^{3} /3M_2.$ From Eq. (5) the factor 8*ϕ* in Eq. (7) is accounting for the thermodynamic nonideality. On the other hand, the $- 6.55$ *ϕ* term contains excluded volume contributions from hydrodynamic sources and is calculated based on pairwise only interactions between the particles, i.e., for very dilute solutions. Motion of the particles alone is responsible for − *ϕ*, and the viscous dragging of fluid surrounding each solvated particle contributes − 4.5*ϕ*. A further contribution of − 1.55*ϕ* reflects the mutual effect of particles on each other, and an additional + 0.5*ϕ* arises from Faxen’s relation that takes into account the non-zero mean value of the second derivative of the fluid viscosity in the environment of any sphere effected by the presence of all others.

An alternative (and more popular) breakdown of contributions to the concentration dependence of *D* was provided by Felderhof (1978), whose expression for Eq. (7) can be written in the form

8 $$D = D_{o} \left[ {1 + \lambda_{T}^{HS} + \left( {\lambda_{o}^{HS} + \lambda_{A}^{HS} + \lambda_{S}^{HS} + \lambda_{D}^{HS} } \right)} \right]\phi = D_{o} \left[ {1 + \left( {\lambda_{T} - \lambda_{H} } \right)\phi } \right]$$

which separates the thermodynamic contribution $\lambda_{T}^{HS}$ from its hydrodynamic counterpart $\lambda_{H}^{HS} .$ As above*,* $D_{o}$ is the diffusion coefficient in the limit of zero solute concentration (*ϕ* → 0) and $\phi$ is the volume fraction of solvated solute, and $\lambda_{T}^{H} \phi$ again equates with the thermodynamic excluded volume [Eq. (5)]. In greater detail, $\lambda_{T}^{HS}$ for uncharged hard spheres with radius $a_{2}$ separated by scalar distance $r$ is given by

9 $$\lambda_{T}^{HS} = \frac{{4{\pi \text{N}}_{{\text{A}}} }}{{M_{2} v_{S} }} \mathop \int \limits_{0}^{{2a_{2} }} r^{2} dr = \frac{3}{{a_{2}^{3} }}\mathop \int \limits_{0}^{{2a_{2} }} r^{2} dr = \, + {8}$$

where the second form of the integral is the expression used by Felderhof (1976). This thermodynamic excluded volume is countered by a hydrodynamic counterpart, the Oseen contribution,

10 $$\lambda_{o}^{HS} = - \frac{3}{{a_{2}^{2} }}\mathop \int \limits_{0}^{{2a_{2} }} rdr = - 6$$

A short-term hydrodynamic interaction term

11 $$\lambda_{A}^{HS} = \frac{3}{{a_{2}^{3} }}\mathop \int \limits_{{2a_{2} }}^{\infty } \left( {\frac{9}{8}\frac{{a_{2}^{6} }}{{r^{4} }} - \frac{5}{4}\frac{{a_{2}^{4} }}{{r^{2} }} + \ldots } \right)dr = - {1}.{831}$$

also provides a counter-contribution, for which the above value (Jones and Schmitz 1988; Cichoki and Felderhof 1988) amends the original estimate of − 1.73 (Felderhof 1978) by taking into account the slow convergence of the series in Eq. (11). These two hydrodynamic terms are opposed by a second short-range interaction

12 $$\lambda_{S}^{HS} = \frac{{75a_{2}^{4} }}{4} \mathop \int \limits_{2a}^{\infty } \frac{{{\text{d}}r}}{{ r^{5} }} = \, + 0.{286}$$

and a dipole force term

13 $$\lambda_{D}^{HS} = \, + {1}$$

The combined hydrodynamic contribution $\lambda_{H}^{HS}$ in Eq. (8) is then 6.546, which supports the value of 6.55 obtained by Batchelor [see Eq. (6)] and also by Wills (1979, 1981), whereupon the dimensionless coefficient for diffusion concentration dependence $\lambda^{HS}$ thus becomes

$$\left( {\lambda_{T}^{HS} - \lambda_{B}^{HS} } \right) = 1.45.$$

It is important to note that this value of $\left( {\lambda_{T}^{HS} - \lambda_{B}^{HS} } \right)$ has been obtained by restricting consideration to the consequences of pairwise solute interactions, which applies to very dilute solutions. A higher value of 3.0 has been derived by Brady and Durlofsky (1988) for higher concentrations, where higher-order terms reflecting multiple-body interactions dominate the extent of nonideality. Indeed, those authors note that their formulation [Eq. (9) of Brady and Durlofsky (1988)] reduces to the Batchelor expression when nonideality is described solely by the consequences of pairwise interactions (*ϕ* → 0) (see also Winzor et al 2021). We continue on the basis that the linear *D–c* dependence for a single solute modeled as an uncharged sphere is reflecting pairwise interactions and therefore described by Eq. (8) with $\left( {\lambda_{T}^{HS} - \lambda_{H}^{HS} } \right) = 1.45.$

### Allowance for effects of solute charge on the concentration dependence of diffusion

As noted by Petsev and Denkov (1992), the presence of net charge $Z_{2}$ on the hard spheres only requires modification of the expressions for $\lambda_{T}$ and $\lambda_{O}$ to accommodate significant additional contributions arising from electrostatic interactions between the two particles. We now summarize the steps involved in the elucidation of those two contributions, which dominate the magnitudes of the dimensionless coefficient *λ*.

The magnitude of the osmotic second virial coefficient for a single solute, $B_{22}$, is given (McMillan and Mayer 1945; Mayer 1950) by

14 $$B_{22} = - 2 \pi N_{A} \mathop \int \limits_{{2a_{2} }}^{{\infty}} f_{22} \left( r \right)r^{2} dr$$

where *r* is the scalar distance between the centers of two solute molecules and where the potential of mean force between them, $u_{22} \left( r \right)$, is contained within the Mayer function:

15 $$f_{22} \left( r \right) = exp\left[ { - \frac{{u_{22} \left( r \right)}}{{k_{B} T}}} \right] - 1$$

For spherical solute molecules with solvated radius $a_{2,}$ the energy function $u_{22} \left( r \right)$ is described in terms of the Debye–Hückel inverse screening length *κ*, the dielectric constant of the solvent medium *ε*, electronic charge *e* and the net protein charge $Z_{2}$ (assumed to be spread uniformly over the spherical surface) by

16 $$\frac{{u_{22} \left( r \right)}}{{k_{B} T}} = \left\{ {\begin{array}{*{20}c} \infty & { r < 2a_{2} } \\ {\frac{{\mathop {Z_{2}^{2} } e^{2} {\text{exp}}\left[ { -\kappa \left( {r - 2a_{2} } \right)} \right]}}{{\varepsilon\left( {1 + \kappa a_{2} } \right)r}}} &  r \ge 2a_{2}   \end{array} } \right.$$

from which it is evident that $f_{22} \left( r \right)$ is − 1 for $r < 2a_{2}$.

A more useful experimental form of Eq. (16) for $r \ge 2a_{2}$ is obtained by taking advantage of the relationship between molar ionic strength $I_{M}$ and inverse screening length,

17 $$\kappa ^2 = \frac{{8\pi \text N_{A} e^{2} I_{M} }}{{\varepsilon{\text{k}}_{{\text{B}}} T}}$$

where upon the potential of mean force for $r \ge 2a_{2}$ becomes

18 $$\frac{{u_{22} \left( r \right)}}{{\text k_{\text B} T}} = \frac{{1000Z_{2}^{2} \kappa^2 \exp [ - \kappa \left( {r - 2a_{2} )} \right]}}{{8\pi \text N_{\text A} I_{M} (1 + \kappa a_{2} )^{2} }}$$

in which the factor of 1000 in the numerator converts the units of ionic strength from molar to the concentration scale (mol/mL) being used here.

From Eq. (14), the expression for the overall thermodynamic excluded volume to the dimensionless coefficient $\lambda_{{{\text{tot}}}}$ is therefore

19 $$\lambda_{T} = \frac{{2B_{22} }}{{M_{2} v_{s} }} = \frac{{4\pi {\text{N}}_{{\text{A}}} }}{{M_{2} v_{s} }}\left[ {\mathop \int \limits_{0}^{{2a_{2} }} r^{2} dr + \mathop \int \limits_{{2a_{2} }}^{\infty } f_{22} \left( r \right) r^{2} dr} \right]$$

in which the first and second integrals refer to $\lambda_{T}^{HS}$ and $\lambda_{T}^{EL} ,$ respectively. Consistency with Eq. (9) is evident on noting that $4\pi \text N_{\text A} /\left( {M_{2} v_{s} } \right) = 3/a_{2}^{3}$. In the same terminology, the complete expression for the Oseen hydrodynamic term (Petsev and Denkov 1992) is

20 $$\lambda_{o} = - \frac{{4\pi \text N_{\text A} a_{2} }}{{M_{2} v_{s} }}\left[ {\mathop \int \limits_{0}^{{2a_{2} }} rdr + \mathop \int \limits_{{2a_{2} }}^{\infty } f_{22} \left( r \right) rdr} \right]$$

which necessarily yields the earlier value of -6 for $\lambda_{o}^{HS}$ [see Eq. (10)]: the second term will be regarded as the negative $\lambda_{H}^{EL}$ contribution.

After allowance for the effects of charge–charge repulsion on excluded volume, the Batchelor expression for the concentration dependence of *D*, Eq. (6), becomes

21 $$D = D_{o} \left[ {1 + \lambda_{tot} \phi } \right]$$

where

22 $$\lambda_{tot} = \left( {\lambda_{T}^{HS} - \lambda_{H}^{HS} } \right) + \left( {\lambda_{T}^{EL} - \lambda_{H}^{EL} } \right) = 1.45 + \lambda_{T}^{EL} - \lambda_{H}^{EL}$$

A similar adjustment would be necessary for the Brady–Durlofsky expression for the concentration dependence of *D* for higher concentration systems (Brady and Durolfsky 1988; Winzor et al 2021).

**Fig.1:**
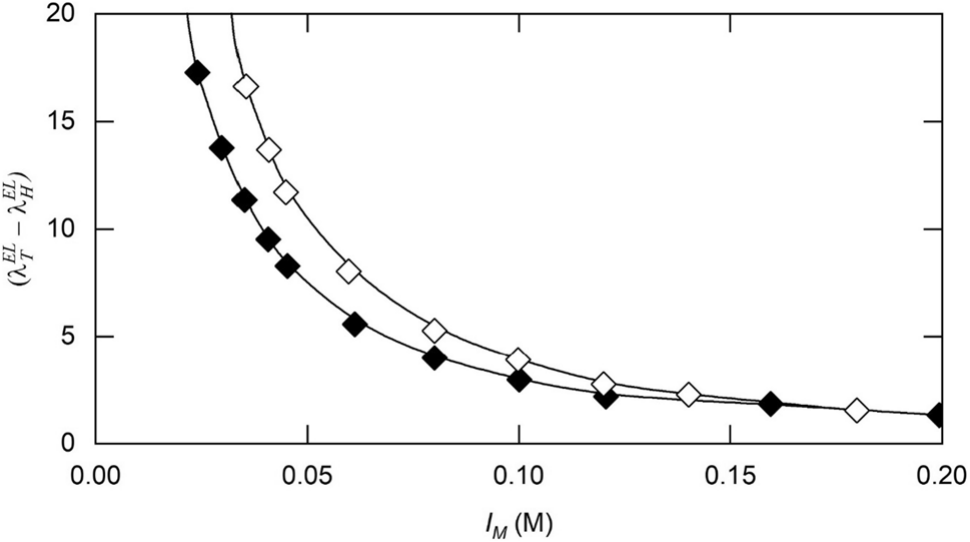
Predicted effects of ionic strength on the electrostatic contribution, ($\lambda_{T}^{EL} - \lambda_{H}^{EL} ),$ to *D–c* dependence for a protein with the size and charge characteristics of bovine serum albumin at neutral pH ($M_{2}$ = 66 kDa, $Z_{2}$ = –20) with a Stokes radius $a_{2}$ of 3.5 nm, a net charge $Z_{2}$ of − 20 and a molecular weight $M_{2}$ of 66,000 based on the exact (♦) – Eq. 22 and approximate (◊)—Eq. 23 forms of Eq. (22)

Values of $\lambda_{T}^{EL}$ and $\lambda_{H}^{EL}$ are most accurately obtained by numerical integration to solve the respective integrals in Eqs. (19) and (20) for specific systems with assigned values of protein radius ($a_{2}$) and net charge ($Z_{2}$) as well as the ionic strength $I_{M}$ of the medium: the value of *κ* (cm^−1^) is obtained as $3.27 \times10^{7} \sqrt {I_{M} }$. The initial treatment of the problem (Petsev and Denkov 1992) incorporates the assumption that ${\text{exp}}\left[ { - u_{22}^{EL} \left( r \right)/(\text k_{\text B} T)} \right]$ ≈ $1 -$ $u_{22}^{EL} \left( r \right)/(\text k_{\text B} T)$, which gives rise to the expression

23 $$\lambda_{tot} = \left( {1.45 + \frac{{1000Z_{2}^{2} }}{{2I_{M} \left( {1 + \kappa a_{2} } \right)M_{2} v_{s} }}} \right)$$

as the approximate counterpart of Eq. (22). However, its use to define the magnitude of $\left( {\lambda_{T}^{EL} - \lambda_{H}^{EL} } \right)$ leads to overestimation of the electrostatic contribution to $\lambda_{tot}$ at low ionic strengths. The extent of the disparity between the correct and approximate estimates of $(\lambda_{T}^{EL} - \lambda_{H}^{EL} )$ is evident from Fig. 1, which summarizes the effect of ionic strength on that concentration dependence coefficient for a protein with the size and charge characteristics of bovine serum albumin at neutral pH ($a_{2}$ = 3.5 nm, $M_{2}$ = 66 kDa, $Z_{2}$ = -20). In keeping with the earlierobservations on osmotic second virial coefficients [see Table 1 of Wills and Winzor (2009)], the approximate values (◊) overestimate their counterparts deduced by numerical integration (♦) by an extent that decreases with increasing ionic strength. Adoption of the numerical integration approach avoids the need for any concern about the evaluated $\lambda_{tot}$ and is accordingly the method adopted in the following considerations of experimental systems.

We now need to establish the relationship between $\lambda _{tot}$ and $k_{D}$, the coefficient for the dependence of $D$ upon solute concentration $c_{2}$. On the grounds that $\phi = v_{s} c_{2}$, it has become standard practice to regard $k_{D}$ as $\lambda_{tot} v_{s}$. However, reference to Eq. (5) reveals that the coefficient describing the thermodynamic excluded volume is $2B_{2} M_{2}$. Obligatory adoption of the same convention for the hydrodynamic and hence combined exclusion volumes leads to the conclusion that

24 $$k_{D} = \lambda_{tot} v_{s} /2 = [(0.725 + \left( {\lambda_{T}^{EL} - \lambda_{H}^{EL} } \right)/2]v_{s}$$

At this stage, no account has been taken of the effect of solution viscosity on the magnitude of the measured diffusion coefficient at finite protein concentrations because Eqs. (7) and (8) imply constancy of the viscosity term *η*. This situation is rectified by writing the concentration dependence of the diffusion coefficient as (Scott et al. 2014)

25 $$D = D_{o} \frac{{1 + k_{D} c_{2} }}{{\eta /\eta _{b} }}$$

where *η* denotes the viscosity of a protein solution with concentration $c_{2}$ for which *D* was measured, and $\eta _{b}$ that of buffer (the solution viscosity in the limit of zero solute concentration to which $D_{o}$ refers). Upon replacement of the relative viscosity of dilute solutions of hard spheres by the expression (Scott et al 2014)

26 $$\eta /\eta_{b} = 1 + [\eta ]c_{2} + \, \cdots \approx 1 + 2.5v_{S} c_{2}$$

Equation (24) becomes

27 $$k_{D} = \left[ { - 1.775 + \left( {\lambda_{T}^{EL} - \lambda_{H}^{EL} } \right)/2} \right]v_{s}$$

which predicts negative *D*–*c* dependence for an isoelectric protein ($$Z_{2} = 0)$$. On the other hand, the sign of $$k_{D}$$ for a charged protein depends upon the extent to which the positively charged term counteracts the negative hard-sphere repulsion term.

### Consideration of results from classical diffusion studies

The availability of a quantitative description of the concentration dependence coefficient $k_{D}$ governing the diffusion coefficient obtained for charged globular proteins under the thermodynamic constraints of constant temperature and solvent chemical potential allows more stringent scrutiny of the extent of conformity between experimental observation and theoretical prediction. For that endeavor, we need to go back to the 1950s to find traditional diffusion experiments involving the spreading of an initially sharp boundary between protein solution and diffusate—an era when the accuracy of diffusion coefficient measurement attained its peak (Gosting 1956). Soon thereafter, the introduction of gel electrophoretic techniques for protein separation led to a loss of interest in moving boundary electrophoresis (Tiselius 1937), the U-tube cell assembly of which was central to those diffusion measurements.

This section begins with the refinement of a previous analysis of diffusion results for ovalbumin and bovine serum albumin (Scott et al. 2014), which was based on the revised value (Brady and Durlofsky 1988) of 5.0*ϕ* (cf 6.55 *ϕ*) for the hydrodynamic term in Eq. (7).

### Traditional diffusion studies of ovalbumin and bovine serum albumin

**Fig. 2:**
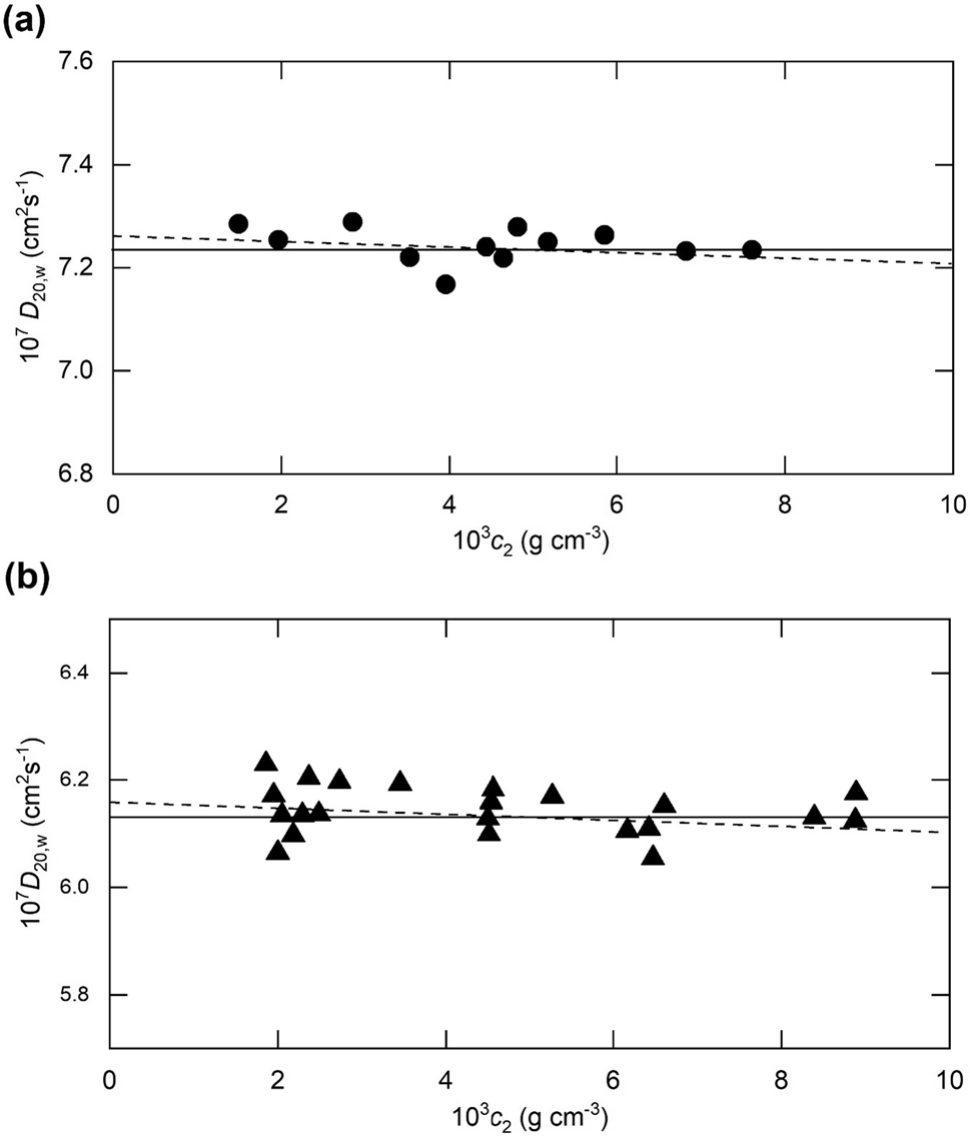
Experimentally measured concentration dependence of the translational diffusion coefficients ($$D_{20,w} )$$ for proteins by the traditional boundary spreading procedure. **a** Ovalbumin in acetate–chloride buffer (pH 4.59, *I* 0.16 M). **b** Bovine serum albumin in phosphate buffer (pH 6.8, *I* 0.10 M). For both proteins, the broken line is the theoretical *D–c* dependence. [Data in (**a)** and (**b)** taken from Creeth et al. (1958) and Creeth (1952), respectively.]

Measurements (Creeth et al. 1958) of the diffusion coefficient (expressed as $D_{20,w}$) for isoelectric ovalbumin (pH 4.59, *I* 0.16 M) are shown (●) in Fig. 2a, which signifies a mean diffusion coefficient of 7.24 × $10^{ - 7}$ cm^2^ s^−1^. It remains to calculate the theoretical dependence predicted by Eq. (27) with $\lambda_{T}^{EL} - \lambda_{H}^{EL} = 0$. Combination of the Stokes radius of 2.9 nm deduced from Eq. (6) with the molar mass of 44 kDa for this glycoprotein (Hall et al. 1999) yields a solvated specific volume ($v_{s}$) of 1.40 mL/g, a limiting diffusion coefficient $D_{o}$ of 7.26 cm^2^s^−1^ and a $k_{D}$ of − 2.5 mL/g that refers to $c_{2}$/2, the mean concentration across the diffusing boundary (Gosting 1956). In that regard, a similar estimate (− 2.0 mL/g) for the concentration coefficient is obtained by linear least squares analysis of the experimental results, but the uncertainty (± 2SD) of the estimate (− 2.6 mL/g) exceeds its absolute magnitude. On the grounds that the same situation applies to the predicted *D*–*c* dependence, we conclude that the most appropriate experimental description of the results is in terms of a concentration-independent $D_{20,w}$ (± 2SD) of 7.24 ($\pm 0.10)\times 10^{ - 7}$ cm^2^ s^−1^ (the solid line in Fig. 2a). Indeed, the absence of any detectable deviation from a Gaussian distribution in a test of boundary skewness in the experiment with highest ovalbumin concentration [Fig. 5 of Creeth et al. 1958)] provides further justification for considering *D* to be effectively concentration independent.

The set of results (▲) in Fig. 2b refers to diffusion measurements (Creeth 1952) on bovine serum albumin in phosphate buffer (pH 6.8, *I* 0.10 M), conditions under which the protein bears a net charge in the vicinity of − 20 (Tanford et al. 1955). In the absence of any discernible concentration dependence, the mean value of (6.14 ± 0.04) × 10^–7^ cm^2^ s^−1^ for $D_{20,w}$ has been used to calculate a Stokes radius of 3.5 nm and hence a specific solvated volume ($v_{S}$) of 1.64 ml/g. For this system, the predicted value of $k_{D}$ is − 1.9 mL/g, whereupon the best-fit description becomes $10^{7} \times D = 6.16\left( {1 - 1.9c_{2} /2} \right)$ cm^2^ s^−1^ (broken line in Fig, 2b). However, that predicted extent of concentration dependence is again smaller than the experimental uncertainty inherent in the diffusion coefficient measurements—a situation that also applies to its slightly negative experimental counterpart ($k_{D}$ = − 1.8 (± 2.6) mL/g). As with ovalbumin (Fig. 2a), the relatively low serum albumin concentrations employed in traditional diffusion experiments ensure that the predicted *D*–*c* dependence is contained within the scatter envelope of measured diffusion coefficients (Fig. 2b).

The above considerations have provided additional theoretical justification for the accepted viewpoint in the 1950s that the translational diffusion coefficient for globular proteins exhibits slight concentration dependence, but that the variation in *D* over the concentration ranges being employed in those days (0.001–0.015 g/mL) was within the uncertainty limits of experimental diffusion coefficient measurements.

### Concentration dependence of *D* from dynamic light scattering measurements

Interpretation of the concentration dependence of diffusion coefficients obtained by dynamic light scattering has invariably been based on single-solute theory. Such action overlooks the need to regard buffer components as additional cosolutes to accommodate the fact that thermodynamic activity is monitored on a molal-type scale (g solute/g solvent) under the operative constraints of constant temperature and pressure (Kirkwood and Goldberg 1950; Hill 1959). However, disregard of the consequences of these protein–cosolute interactions seems to have little impact on the predicted magnitudes of thermodynamic excluded volume contributions to nonideality (Winzor et al. 2007). We therefore persist with the description of dynamic light scattering measurements in terms of single-solute theory, but do take into account the difference between molar ($$B_{22} /M_{2} )$$ and molal ($$C_{22} /M_{2} )$$ second virial coefficients. Subject to the assumed incompressibility of aqueous solutions, the two virial coefficients are related by the expression (Wills et al. 1993)

28 $$C_{22} /M_{2} = (B_{22} /M_{2} - \overline{v}_{2} { })\rho_{1}$$

where $\overline{v}_{2}$ is the anhydrous partial specific volume of the solute and where $\rho_{1} ,$ the solvent density, has been taken as 1 g/mL for water.

In addition, Phillies (1987, 1992) has challenged the use of 1.45 for $\left( {\lambda_{T}^{HS} - \lambda_{H}^{HS} } \right)$ in the interpretation of dynamic light scattering measurements on the grounds that light scattering spectroscopy is sensitive to particle position, whereas the Batchelor/Felderhof treatment refers to particle flux effected by a gradient in solute chemical potential. Phillies (1987) calculates a value of − 0.9 for $\left( {\lambda_{T}^{HS} - \lambda_{H}^{HS} } \right)$. The modified form of Eq. (27), namely

29 $$k_{D} = \left[ { - 2.95 + \left( {\lambda_{T}^{EL} - \lambda_{H}^{EL} } \right)/2} \right]v_{s } - \overline{v}_{2}$$

incorporates that change in the hard-sphere contribution as well as the fact that the excluded volumes responsible for nonideality in dynamic light scattering are molal volumes being measured experimentally on a molar concentration scale.

### Consideration of experimental dynamic light scattering results

Interest in the determination of $k_{D}$ by dynamic light scattering was fostered by the realization that the return of a slightly negative value provided a potential diagnostic of conditions commensurate with protein crystallization (Eberstein et al. 1994). In those days the quantitative description of the ionic strength dependence of $k_{D}$ also incorporated a curve-fitting parameter (Hamaker 1937) to account for attractive inter-solute forces. On the grounds that the effects of those van der Waals attractive forces are already included in the thermodynamic excluded volume (Wills and Winzor 2005) and presumably the hydrodynamic counterpart, it becomes of interest to examine the extent to which the present approach can predict that experimental ionic strength dependence of $k_{D}$ for lysozyme (Eberstein et al. 1994).

**Fig. 3:**
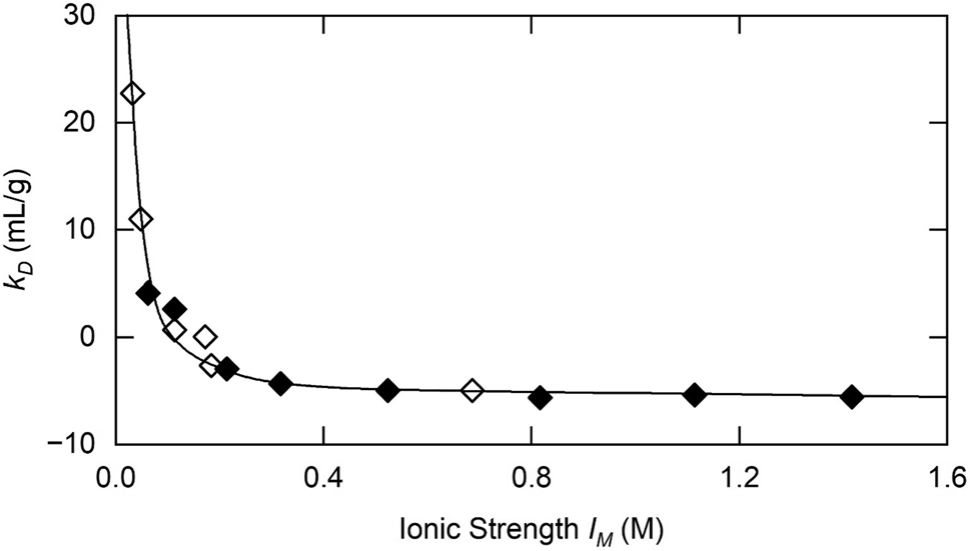
Ionic strength dependence of the concentration coefficient $k_{D}$ for lysozyme at pH 4.2 (♦) and its best-fit description (^_____^) in terms of Eq. (29), together with results (◊) from a study under similar conditions [Based on data from Table 1 of Eberstein et al. (1992) and Table 2 of Muschol and Rosenberger (1995), respectively.]

The theoretical description of the ionic strength dependence of $$k_{D}$$ predicted by Eq. (29) for lysozyme at pH 4.2 (Fig. 3) clearly provides a good description of the results (♦) reported for this enzyme in Table 1 of Eberstein et al, (1994). Their value of 2.09 nm for the Stokes radius (derived from $$D_{o} )$$ was used for $$a_{2}$$, and the valence ($$Z_{2}$$) was taken as + 12, the value inferred from pH-titration data (Tanford and Wagner 1954): the reported value of 0.703 mL/g for lysozyme (Sophianopoulos et al. 1962) was used for $$\overline{v}_{2}$$. Results reported in Table 2 of Muschol and Rosenberger (1995) for lysozyme under similar conditions (◊) are also described adequately by the same theoretical ionic strength dependence. The above value for the net charge seems more realistic than those of 5.4–6.8 (Eberstein et al. 1994; Muschol and Rosenberg 1995; Kuehner et al. 1997) deduced on the basis of $$Z_{2}$$ and the Hamaker constant as curve-fitting parameters from lysozyme studies under similar conditions.

**Fig. 4:**
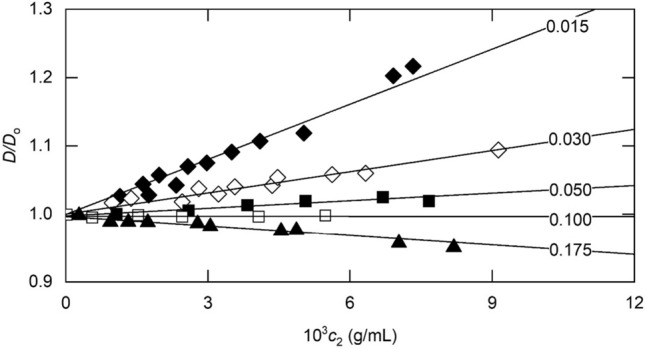
Concentration dependence of the diffusion coefficient for a monoclonal antibody (pH 5) at the indicated molar ionic strengths, together with descriptions in terms of Eq. (29). [Data inferred from Fig. 2 of Arzenšek et al. (2012)]

Further support for the adequacy of Eq. (29) as a theoretical description of diffusion measurements obtained by dynamic light scattering comes from Fig. 4, which summarizes results deduced from Fig. 2 of Arzenšek et al. (2012) for a monoclonal antibody ($M_{2}$ = 145 kDa) at pH 5.75 and molar ionic strengths (*I*_*M*_) of 0.0.015 (♦), 0.030 (◊), 0.050 (■) and 0.175 (□). Lines denote the *D*–$c_{2}$ relationships predicted by Eq. (29) on the basis of the solute radius $a_{2}$ of 5.4 nm that stems from the value of 4.49 cm^2^ sec^−1^ for $D_{o}$: $\overline{v}_{2}$ was taken as 0.73 mL/g. The value of 75 for $Z_{2}$, deduced as a curve-fitting parameter, is consistent with the estimate of (68 ± 15) obtained from the zeta potential measurements reported in Fig. 8 of Arzenšek et al. (2012). The fact that the present analysis, based solely on excluded volume and solution viscosity considerations, has yielded an acceptable description of the experimental data calls into question the need (Arzenšek et al. 2012) for incorporation of the Hamaker constant as an additional curve-fitting parameter to cover the consequences of solute–solute interactions. Indeed, it can be argued that the current agreement between experimental and predicted dependencies of $k_{D}$ upon ionic strength supports the contentionthat those van der Waals interactions are already included not only in the thermodynamic excluded volume (Wills and Winzor 2005) but also in its hydrodynamic counterpart, an assumption inherent in the present analysis.

## Concluding remarks

The first objective of this review has been to justify consideration of the translational diffusion coefficient for globular proteins as a constant for the analysis of boundary spreading in experiments conducted under the thermodynamic constraints of constant temperature and solvent chemical potential. To that end the Batchelor treatment of *D*–*c* dependence for an uncharged spherical particle (Batchelor 1976) used previously (Scott et al. 2014) has been extended by incorporating relevant quantitative expressions from the dynamic light scattering literature (Petsev and Denkov 1992; Arzenšek et al. 2012) that include the effects of net charge on a spherical particle. The slightly positive *D*–*c* dependence thereby predicted for an isoelectric protein [Eq. (22)] becomes negative after allowance is made (Scott et al. 2014) for the effect of solution viscosity [Eqs. (25)–(27)]. However, because the extent of experimental uncertainty in diffusion coefficient measurements (> ± 1%) exceeds that of the predicted concentration dependence of *D*, the appropriate course of action is to regard the diffusion coefficient as a constant in the analysis of boundary spreading in sedimentation velocity distributions for globular proteins obtained in the heyday of the model E ultracentrifuge (Baldwin 1957; Fujita 1956, 1959; Van Holde 1960; Creeth and Winzor 1962). A much later example supporting this contention is the SEDFIT analysis of sedimentation velocity distributions for a 12 g/L solution of halophilic malate dehydrogenase (pH 8, $I_{M}$ 4.0), where allowance for *D*–*c* dependence led to no improvement in the best-fit description (Solovyova et al. 2001).

That simplification of the analysis of boundary spreading does not apply to sedimentation velocity experiments on proteins in media with very low ionic strength because of the highly positive value of $k_{D }$ [Fig. 1 and Eq. (27)]. Nor does it apply to the sedimentation of concentrated protein solutions at moderate ionic strengths, where the magnitude of $k_{D} c_{2}$ can become significant despite the relatively small (negative) magnitude of the diffusion concentration coefficient. Examples of these two situations are provided by studies of (i) a monoclonal antibody (10 g/L) in 2 mM histidine buffer, pH 6, and (ii) bovine serum albumin (52 g/L) in phosphate-buffered-saline, pH 7.4 [Figs. 1 and 5, respectively, of Chaturvedi et al. (2018)].

A potential outcome of attempted simultaneous evaluations of the sedimentation concentration dependence parameter $k_{s}$ and $k_{D}$ stems from the inevitable correlation between estimates of the two curve-fitting parameters. For example, the return of a $k_{D}$ estimate of + 10 mL/g for bovine serum albumin in phosphate-buffered-saline [cf a calculated value of -2.9 mL/g from Eq. (27)] has led to a correlated estimate of 8.4 mL/g for $k_{s}$ that is larger than its independently determined counterpart of 7.2 mL/g [Fig. 8 of Winzor et al. (2021)]. In other words, the greater extent of diffusional boundary spreading generated by the curve-fitting overestimate of $k_{D}$ has been compensated by an increase in $k_{s}$ to achieve the extra boundary sharpening required to maintain the experimental boundary shape. Removal of the interdependence of $k_{s}$ and $k_{D}$ estimates by incorporating an experimentally measured value of the sedimentation concentration coefficient should in our opinion lead to improved delineation of $k_{D}$ from the $c_{NI} \left( {s_{o} } \right)$ approach developed by Chaturvedi et al. (2018).

The second objective of this investigation has been the adaptation of the theoretical description of *D*–*c* dependence in traditional diffusion experiments [Eq. (27)] to encompass measurements of diffusion coefficients by dynamic light scattering. Despite the change of thermodynamic constraint from constant solvent chemical potential to constant pressure, a slightly modified version of the same quantitative expression [Eq. (29)] has provided good descriptions of dynamic light scattering data for lysozyme (Fig. 3) and a monoclonal antibody (Fig. 4), chosen because of the ready availability of the data.

The seeming disagreement about the need for allowance for the concentration dependence of *D* has thus merely reflected the protein concentration range over which diffusion coefficients were measured by the two techniques. In the traditional procedure, the resolving power of the optical systems sufficed for the accurate specification of boundary spreading across boundaries a concentration difference in the range 1–10 g/L. On the grounds that the measured *D* refers to $c_{2} /2$ (Gosting 1956), the uppermost concentration covered by the $D - c$ dependence was only ~ 5 g/L in diffusion and sedimentation velocity experiments with the boundary between solution and diffusate. Nevertheless, the accuracy of those diffusion coefficients still sufficed for detection of the slight negative *D–c* dependence even though experimental scatter also justified its consideration in terms of concentration-independent diffusion (Fig. 2a, b).

In dynamic light scattering, the lesser sensitivity of the detection system dictates the use of higher protein concentrations for accurate definition of the autocorrelation function *g*(τ) (see, for example, Harding et al. 1992 and references therein), where τ is the delay time, particularly in studies of small proteins such as lysozyme for which concentrations as high as 60 g/L have been employed to define the *D–c* dependence (Eberstein et al. 1994; Muschol and Rosenberger 1995). The slower diffusion of larger proteins allows more accurate delineation of *g*(τ) and hence the use of a smaller concentration range for more definitive characterization of the *D–c* dependence (Fig. 4).

Finally, the current quantitative treatment of concentration-dependent diffusion based solely on excluded volume and solution viscosity considerations differs from its predecessors in that no specific allowance has been made for the consequences of solute–solute interaction, namely the “*a”* term in the equation of state for an imperfect gas (van der Waals 1873). As noted previously (Wills and Winzor 2005), the thermodynamic consequences of those interactions are already included in the osmotic second virial coefficient contribution, $\left( {8 + \lambda_{T}^{EL} } \right)v_{s}$ in the present context. Based on the adequacy of the predicted descriptions of *D–c* dependence by Eq. (27) or Eq. (29) for the present systems (Figs. 2, 3, 4), it would seem that the hydrodynamic consequences of van der Waals interactions are contained with the corresponding excluded volume term, $(\lambda_{H}^{HS} - \lambda_{H}^{EL} )$. Indeed, the inclusion of $\lambda_{A}^{HS}$ [Eq. (10)] as a short-range interaction contribution to the hard-sphere hydrodynamic term (-6.55 for systems studied under the constraints of constant temperature and solvent ionic strength) supports that contention.

At this stage, the parameter requiring further investigation is the value of the hard-sphere contribution, $\left( {\lambda_{T}^{HS} - \lambda_{H}^{HS} } \right)$, to be used for the prediction of *D*–*c* dependence in dynamic light scattering studies of protein solutions. We have employed the reported value of –0.9 for spheres of (Phillies 1987), which takes into account the non-compliance of experimental conditions (constant *T*, $\mu_{1}$) to which the commonly used value of + 1.45 (Batchelor 1976; Felderhof 1978) applies. The only previous experimental support for adopting the lower value has come from dynamic light scattering studies of stearylated silicon spheres in non-aqueous solvents (Mos et al. 1986). This aspect of the quantitative interpretation to be placed on the *D*–*c* dependence obtained by dynamic light scattering would clearly benefit from further investigation with a range of well-characterized globular proteins and other macromolecular assemblies. It has to be born mind also the assumption of hard spheres commonplace amongst discussions. Deviations in this behavior for non-spherical particles have to be taken into consideration, particularly because of the sensitivity of solution viscosity to conformation: this is discussed further in Winzor et al (2023).
